# Impact of multiparametric magnetic resonance imaging targeted biopsy on functional outcomes in patients following robot-assisted laparoscopic radical prostatectomy

**DOI:** 10.3389/fsurg.2023.1305365

**Published:** 2023-11-20

**Authors:** Conrad Leitsmann, Annemarie Uhlig, Felix Bremmer, Mirjam Naomi Mohr, Lutz Trojan, Marianne Leitsmann, Mathias Reichert

**Affiliations:** ^1^ Department of Urology, University Medical Center Goettingen, Goettingen, Germany; ^2^ Department of Pathology, University Medical Center Goettingen, Goettingen, Germany; ^3^ Department of Urology, Medical University of Graz, Graz, Austria

**Keywords:** prostate cancer, localized, mpMRI-guided prostate biopsy, frozen section, nerve-sparing (NeuroSAFE), secondary resection, robot-assisted laparoscopic radical prostatectomy (RALP), EPIC 26

## Abstract

**Introduction:**

Multiparametric magnetic resonance imaging guided prostate biopsy (mpMRI PBx) leads to a higher rate of successful nerve-sparing in robot-assisted laparoscopic prostatectomy (ns-RALP) for prostate cancer (PCa). This study aimed to evaluate the impact of mpMRI PBx compared to standard ultrasound-guided PBx on functional outcomes focusing on erectile function in patients following ns-RALP.

**Material and methods:**

All RALPs performed between 01/2016 and 06/2021 were retrospectively stratified according to (attempted) ns vs. non ns RALPs and were then categorized based on the PBx technique (mpMRI PBx vs. standard PBx). We compared RALP outcomes such as pathological tumor stage, rates of secondary nerve resection (SNR) and positive surgical margin status (PSM). Furthermore, we explored the association between PBx-technique and patient-reported outcomes assessed 12 months after RALP using the prospectively collected 26-item Expanded Prostate Cancer Index Composite (EPIC-26) questionnaire. Chi-square tests and logistic regression analysis were conducted.

**Results:**

A total of 849 RALPs included 517 (61%) procedures with (attempted) ns. Among these, 37.5% were diagnosed via preoperative mpMRI PBx. Patients with a preoperative standard PBx had a 57% higher association of PSM (*p* = 0.030) compared to patients with mpMRI PBx and a 24% higher risk of erectile dysfunction (ED) 12 months post RALP (*p* = 0.025). When ns was attempted, we observed a significantly higher rate of SNR in patients who underwent a standard PBx compared to those who received a mpMRI PBx (50.8% vs. 26.7%, *p* < 0.001) prior RALP. In comparison, upgrading occurred more often in the standard PBx group (50% vs. 40% mpMRI PBx, *p* = 0.008).

**Conclusion:**

The combination of mpMRI PBx for PCa diagnosis followed by ns-RALP resulted in significantly fewer cases of SNR, better oncological outcomes and reduced incidence of ED 1 year after surgery. This included fewer PSM and a lower rate of postoperative tumor upgrading.

## Introduction

Preservation of neurovascular bundle during nerve-sparing open and robot-assisted laparoscopic radical prostatectomy (ns-RALP) for prostate cancer (PCa) is proven to be associated with a better erectile function and a higher rate of urinary continence following surgery (1–3). Several techniques for nerve-sparing approaches have been developed and the “best” way is still to be found. Nevertheless, NeuroSAFE frozen section, firstly described by Schlomm et al. (4), results in multiple advantages such as less positive surgical margins (PSM) (4, 5) (and higher rates of successful nerve-sparing (4) without affecting oncological outcomes (6).

National and international guidelines advocate for the use of multiparametric magnetic resonance imaging (mpMRI) of the prostate prior to prostate biopsy (PBx) (7–10). The use of mpMRI targeted PBx has relevantly improved the detection of clinically significant PCa defined as International Society of Urological Pathology (ISUP) grade ≥2 (Gleason grade ≥7a) compared to standard ultrasound guided PBx (11, 12). We previously showed, that mpMRI PBx prior RALP was further associated with a higher rate of successful ns and less secondary nerve resection (SNR) compared to standard PBx (13).

Our finding suggested that preoperative imaging and biopsy technique might also affect functional outcomes (13). We hypothesized that patients could experience improved preservation of erectile function if they receive mpMRI PBx before undergoing RALP. Given the increasing importance of patient reported outcome measurements (PROMs) in combination with clinical parameters, erectile function is best measured using a self-reported questionnaire such as the “26-item Expanded Prostate Cancer Index Composite (EPIC 26)” (14, 15).

The aim of this study was to evaluate the potential benefit of mpMRI PBx over standard PBx on erectile function 12 months after RALP.

## Materials and methods

### Study population

Based on an institutional ethics board approval, our institution prospectively collects data of all patients with PCa who undergo RALP. The current study includes all consenting patients, who underwent RALP between January 2016 and June 2021. We analyzed a range of clinical, perioperative, and oncological data, including age, initial prostate specific antigen value at diagnosis (iPSA), result of digital rectal examination (DRE), initial Gleason score/ISUP 2014 grade, PBx technique, the success/failure of ns and rate of SNR, operation time (measured from urinary bladder catheter placement to last skin stitch). The study was approved by the local Ethics Committee of the University Medical Center Göttingen.

### Prostate biopsy techniques

Patients included in the study had undergone either systematic transrectal ultrasound-guided “standard” PBx or perineal systematic as well as targeted mpMRI PBx. PBx was typically performed by the treating outpatient urologist, while mpMRI PBx was mainly conducted at our institution. Patients undergoing PBx did not receive mpMRI prior to biopsy. The PBx procedure involved taking 10–12 biopsy cores of the prostate using transrectal ultrasound. In our clinic, we no longer perform standard transrectal systematic biopsies, like outpatient urologists do. Therefore, 100% of the patients in the standard PBx group were treated there. In contrast, all patients who underwent mpMRI PBx received a standardized mpMRI scan using the Prostate Imaging Reporting and Data System (PI-RADS) version 2.0. All mpMRI reports were interpreted by specialized and trained radiologists. Using the PI-RADS 2.0 classification, all PIRADS ≥3 lesions were targeted with 4–5 biopsies per lesion, in addition to a systematic biopsy taking between 10 and 20 cores. MpMRI PBx was performed perineally using Biopsee© (Fa. MedCom, Darmstadt, Germany) (16).

### Robot-assisted laparoscopic radical prostatectomy (RALP) and NeuroSAFE

A transabdominal RALP with pelvic lymph node dissection (LAD) was performed in all patients using either the Da Vinci System Si© or Da Vinci System Xi© (Intuitive Surgical, Sunnyvale, CA, USA) (17). The surgical techniques, including the preservation and reconstruction of the pelvic floor, were standardized in order to ensure consistency and comparability across patients (18). In specific circumstances, such as when younger patients expressed a strong preference for a nerve-sparing approach despite having a high-risk oncological context, a personalized treatment pathway was followed and a ns-RALP was performed.

Preservation of the neurovascular bundle was carried out whenever it was oncologically feasible according to the guidelines (≤cT2) and intraoperative findings, and the patient expressed a preference for it (9). The patient's request for nerve preservation was recorded during a preoperative discussion. For oncological safety we performed a frozen section of the entire dorsolateral part of the gland surfacing the neurovascular bundle (from urethra to the bladder neck) during RALP (NeuroSAFE). When there was a cancer-positive area of the surgical margin (iopPSM), the corresponding bundle was fully resected. Intra-fascial NS approach was performed in all (attempted) ns RALPs as described by Budäus et al. for the open approach (19).

### Oncological outcomes

We evaluated postoperative cancer-related outcomes such as the postoperative Gleason score/ISUP 2014 grade and PSM based on the final pathology sample, potential oncological upgrading of the tumor stage, and nodal stage as per the LAD specimen.

### Patient reported outcomes

Patients were asked to answer the questions of the fifth version of the EPIC-26 just before and 12 months after undergoing RALP. The EPIC-26 questionnaire consists of five domains: urinary incontinence, urinary irritative/obstructive symptoms, hormonal function, gastrointestinal symptoms, and sexuality. All domains have a point range from 0 to 100, with less points indicating lower function. Scoring of the answers given by the patients was calculated according to standardized scoring instructions (20). Primary endpoint of this study was post-RALP Sexual Summary Score (SexSS) of the EPIC-26 ranging between 0% (worst) to 100% (best). ED was defined by the frequency of erections (EPIC-26 item 10: ≤“I had an erection less the half the time I wanted one”).

### Statistical analysis

First, the total study population was divided into two groups based on whether they underwent (attempted) ns-RALP or not. Subsequently, patients who underwent ns-RALP were further split based on the biopsy technique (PBx vs. mpMRI PBx).

Continuous measures were summarized using means and standard deviations or medians and interquartile ranges, depending on the distribution of the data. Categorical data were presented as absolute numbers and percentages. Statistical analyses were conducted using either Student's *t*-tests or Mann Whitney *U* tests for continuous variables, depending on the data distribution. We used Chi-square for categorical variables. Chi-square and multivariable logistic regression analyses were used for prediction ED.

We also studied time trends and changes in the application of mpMRI PBx and NeuroSAFE technique. For both purposes, we used the Jonckheere-Terpstra test.

The significance level was chosen at *p* < 0.05. All analyses were performed using Statistical Package for the Social Sciences (SPSS, Inc., Chicago, IL, version 28).

## Results

### Patients' characteristics of the total cohort and stratified according to PBX technique

**Figure 1:**
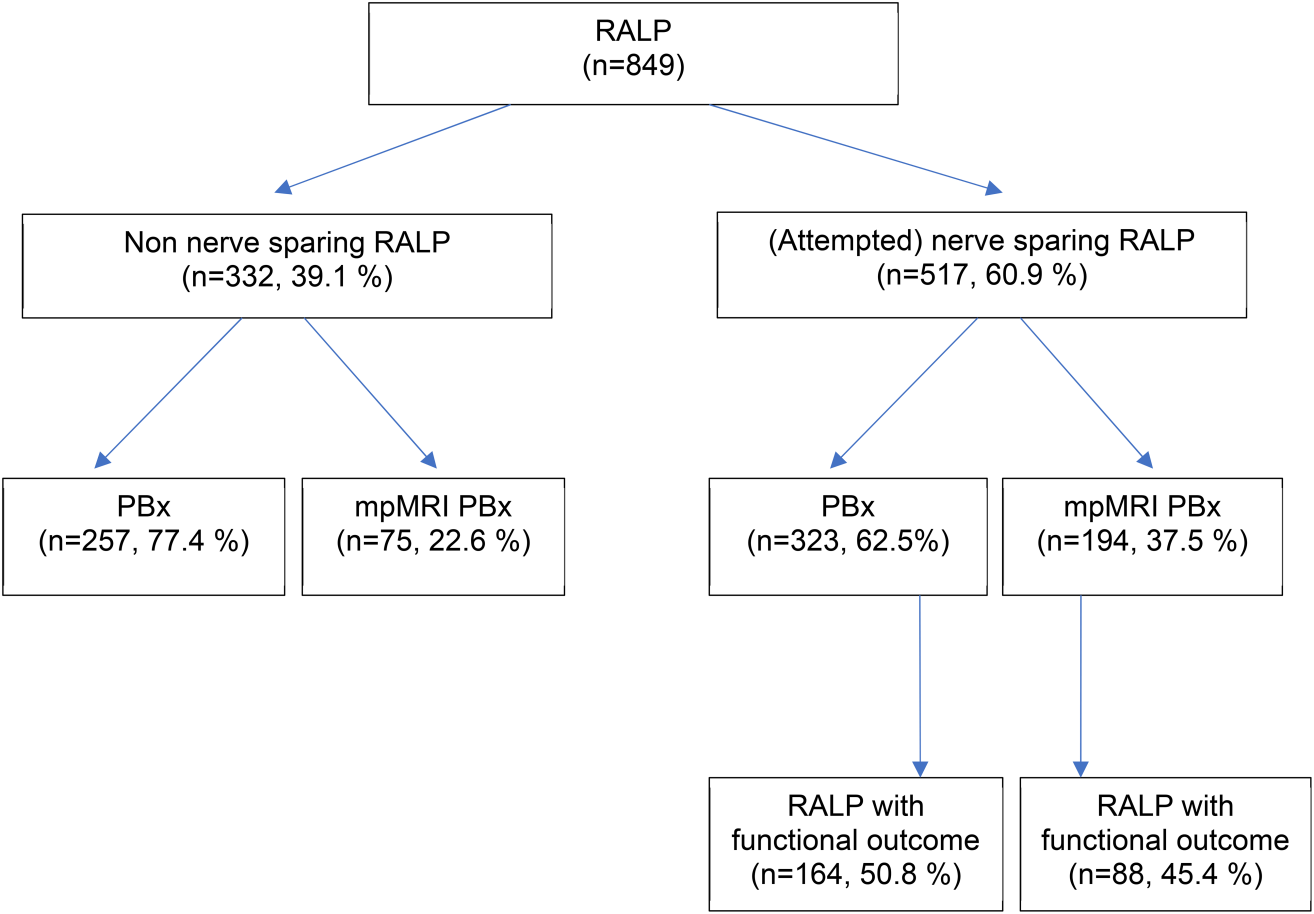
Study population. RALP, radical prostatectomy; PBx, ultrasound (US)-guided prostate biopsy, mpMRI PBx, mpMRI (multiparametric magnetic resonance imaging) targeted prostate biopsy.

Our study included a total of 849 patients who underwent RALP for PCa. The mean age of the total cohort was 66 years. 517 patients underwent (attempted) ns-RALP and 332 patients were scheduled for a non ns approach (Figure 1). iPSA value did not differ in both biopsy groups (12 vs. 12.5 ng/ml, *p* = 0.35). Nevertheless, patients with a standard PBx had a higher rate of suspicious DRE (38% vs. 23%, *p* < 0.001). The (attempted) ns-RALP subgroup had a higher proportion of patients who underwent preoperative mpMRI PBx (37.5%) compared to the non-ns-RALP group (22.6%, *p* < 0.001) (Figure 1). 72% (194/269) of the patients diagnosed by mpMRI PBx and 55% (323/580) of those diagnosed by standard PBx received an (attempted) ns-RALP (Table 1).

**Table 1:** Preoperative clinical and oncological patient characteristics of the total study cohort (*n* = 849) and the (attempted) nerve sparing RALP group (*n* = 517) stratified according to biopsy technique.

	Total study cohort (*n* = 849)			(Attempted) nerve sparing RALP group (*n* = 517)		
	PBx (*n* = 580)	mpMRI PBx (*n* = 269)	*p*-value	PBx (*n* = 323)	mpMRI PBx (*n* = 194)	*p*-value
Mean Age [years] (±standard deviation)	66 (±6.8)	66.9 (±6.8)	0.056	64 (±6.7)	66.2 (±6.4)	<0.001
Mean prostate size [ml] (±standard deviation)	44.4 (±21.6)	50.7 (±26.6)	<0.001	44 (±20)	51.5 (±27.5)	<0.001
Preoperative Gleason/ISUP grade *n* (%)			0.006			0.879
6/1	130 (22.4)	66 (24.5)		105 (32.5)	58 (30)	
7a/2	216 (37.3)	132 (49.1)		156 (48.3)	101 (52.1)	
7b/3	116 (20)	41 (15.2)		46 (14.2)	24 (12.4)	
8/4	83 (14.3)	20 (7.4)		14 (4.3)	10 (5.2)	
9/5	32 (5.5)	10 (3.7)		2 (0.6)	1 (0.5)	
10/5	3 (0.5)	0		0	0	
Mean initial PSA [ng/ml] (±standard deviation)	12.04 (±16.2)	12.5 (±18.2)	0.35	7.7 (±4.9)	9.5 (±5.7)	0.004
Suspicious DRE (%)	221 (38.1)	63 (23.4)	<0.001	69 (21.4)	38 (19.6)	0.655

RALP, radical prostatectomy; PBx, ultrasound-guided prostate biopsy; mpMRI PBx, mpMRI (multiparametric magnetic resonance imaging) targeted prostate biopsy.

Among patients who underwent (attempted) ns- RALP, 95% were diagnosed with ISUP grade 1–3, in contrast to 63% in the non ns-RALP subgroup. Overall, the (attempted) ns-RALP patients had a lower mean iPSA (8.4 vs. 17 ng/ml, *p* < 0.001) (Table 1).

### Clinical and oncological outcomes

Patients with an (attempted) ns- RALP with a preoperative mpMRI PBx experienced less often SNR than patients with a PBx (26.7% vs. 50.8%, *p* < 0.001) (Table 2).

**Table 2:** Postoperative oncological patient characteristics of the total study cohort (*n* = 849) and the (attempted) nerve sparing RALP group (*n* = 517) stratified according to biopsy.

	Total study cohort (*n* = 849)			(Attempted) nerve sparing RALP group (*n* = 517)		
	PBx (*n* = 580)	mpMRI PBx (*n* = 269)	*p*-value	PBx (*n* = 323)	mpMRI PBx (*n* = 194)	*p*-value
Postoperative Gleason/ISUP grade *n* (%)			<0.001			0.05
6/1	58 (10)	28 (10.4)		49 (15.2)	27 (13.9)	
7a/2	226 (40)	143 (53.2)		163 (50.4)	113 (58.2)	
7b/3	151 (26)	67 (24.9)		78 (24.1)	45 (23.2)	
8/4	68 (11.7)	17 (6.3)		15 (4.6)	7 (3.6)	
9/5	75 (12.9)	13 (4.8)		18 (5.6)	2 (1)	
Nodal positive (%)	41 (7.1)	7 (2.6)	0.01	7 (2.2)	2 (1)	0.494
Positive surgical margin (%)	136 (23.4)	43 (16)	0.014	52 (16.1)	27 (13.9)	0.53
Secondary nerve resection (%)	164 (28.3)	53 (19.7)	0.009	164 (50.8)	52 (26.7)	<0.001
Upgrading (%)	288 (49.7)	107 (39.8)	0.008	153 (47.4)	75 (38.7)	0.05
Surgery time (±standard deviation)	3 h 14 min (±48 min)	3 h 27 min (±51 min)	<0.001	3 h 26 min (±42 min)	3 h 37 min (±45 min)	0.005

RALP, radical prostatectomy; PBx, ultrasound (US)-guided prostate biopsy; mpMRI PBx, mpMRI (multiparametric magnetic resonance imaging) targeted prostate biopsy.

Patients with a preoperative standard PBx had a 57% higher risk of PSM (*p* = 0.030) compared to patients with mpMRI PBx (Table 3).

**Table 3:** Multivariable analysis of predictors for positive surgical margin in the final RALP specimen (*n* = 849).

	*p*-value	odds ratio
Age	0.607	1.007
iPSA	**<0** **.** **001**	1.037
mpMRI PBx	**0** **.** **030**	0.651

mpMRI PBx, mpMRI (multiparametric magnetic resonance imaging) targeted prostate biopsy; iPSA, initial PSA at diagnosis.

Bold values are statistically significant.

**Figure 2:**
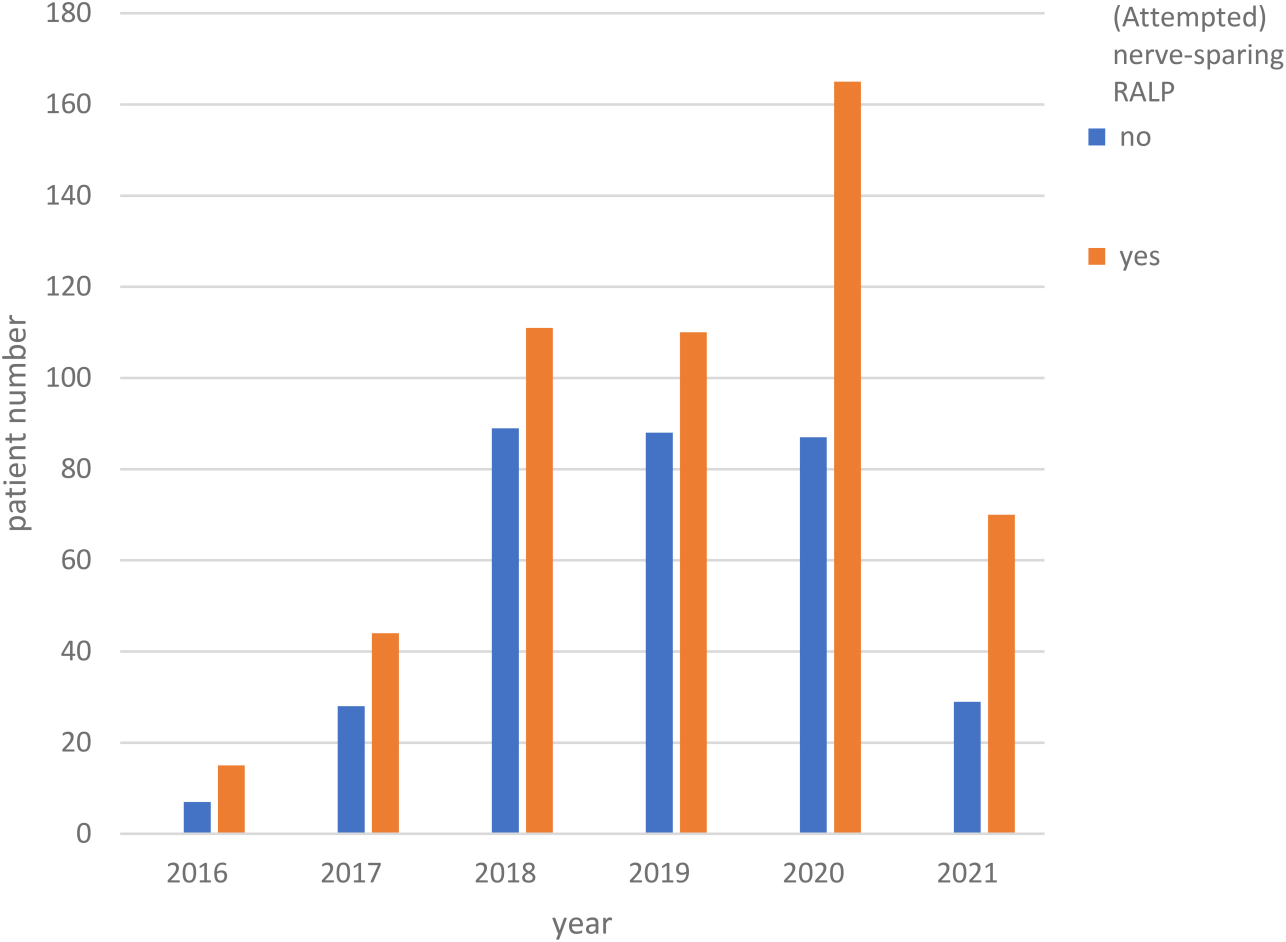
Time trend of nerve sparing RALP over the years (*p* < 0.018). RALP, radical prostatectomy.

**Figure 3:**
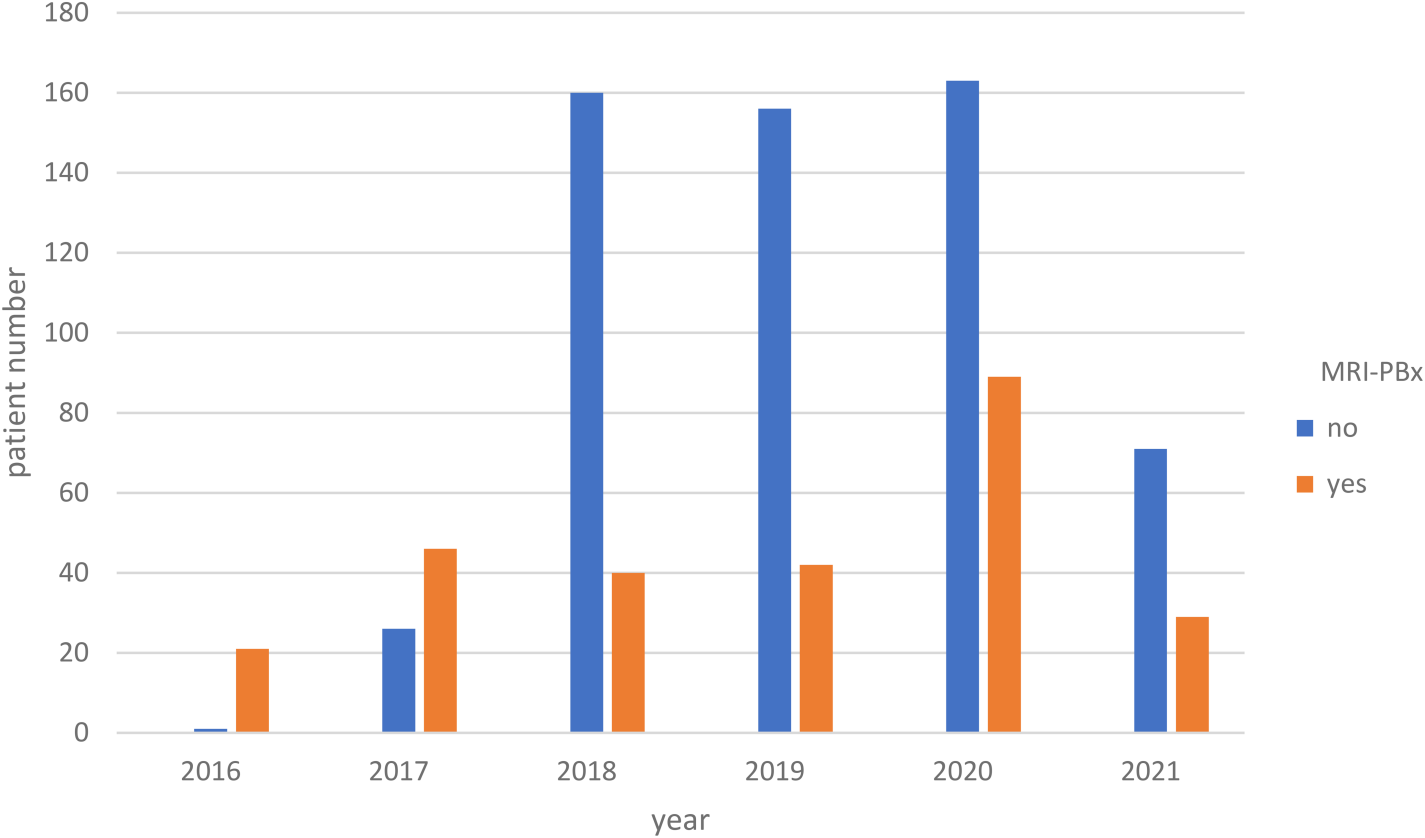
The distribution of preoperative mpMRI PX over the years (*p* < 0.001). mpMRI PBx, mpMRI (multiparametric magnetic resonance imaging) targeted prostate biopsy.

Our analysis revealed a clear trend over the years (2016–2021) indicating a significant increase in nerve preservation among the patients we operated on (*p* < 0.018, Figure 2). Additionally, from 2018 onward, there was a clear trend towards an increase in mpMRI PBx use prior to surgery (*p* < 0.001, Figure 3).

### Functional outcomes

Out of the total of 513 functional evaluations conducted using the EPIC 26 questionnaire, 252 patients after (attempted) ns-RALP were available for analysis of the functional outcomes, including both preoperative and 12-month postoperative assessments. Table 4 shows results of univariate analysis comparing PCa-patients diagnosed with either mpMRI PBx or standard PBx in regard of their functional characteristics. In our univariate analysis none of the parameter of EPIC 26 were significant. In the multivariable analysis mpMR PBx was a significant negative predictor for ED. ED was defined by the frequency of erections (EPIC-26 item 10: ≤“I had an erection less than half the time I wanted one”). Patients with mpMRI PBx had a 24% higher risk of erectile dysfunction (ED) 12 months post RALP (*p* = 0.025) (Table 5).

**Table 4:** Functional outcomes according to the biopsy technique [subgroup with (attempted) nerve sparing RALP, *n* = 252] using EPIC 26 questionnaire.

	Total cohort (*n* = 252)	PBx (*n* = 164)	mpMRI PBx (*n* = 88)	*p*-value
Difference in erectile function (pre to postoperative) (±standard deviation)	−30.3 (±26.7)	−32.7 (±26.6)	−25.9 (±26.6)	0.094
Difference incontinent complaints (pre to postoperative) (±standard deviation)	−16.6 (±27)	−18.2 (±27.3)	−13.8 (±26.5)	0.23
Difference in irritating complaints (pre to postoperative) (±standard deviation)	2.4 (±14.7)	2.2 (±15.9)	2.7 (±12)	0.8
Difference in hormonal complaints (pre to postoperative) (±standard deviation)	6.9 (±22.4)	7.3 (±22.6)	6.3 (±22.1)	0.433
Difference in gastroenterological complaints (pre to postoperative) (±standard deviation)	−3 (±24)	−7.7 (±24.4)	0.5 (±15.1)	0.132
Manifest erectile function (%)	72 (28.6)	50 (30.5)	22 (25)	0.229

PBx, ultrasound-guided prostate biopsy; mpMRI PBx, mpMRI (multiparametric magnetic resonance imaging) targeted prostate biopsy.

**Table 5:** Multivariable analysis of predictors for ED among patients with (attempted) nerve sparing RALP (*n* = 252).

	*p*-value	odds ratio
mpMRI PBx	**0** **.** **025**	0.464
Suspicious DRE	0.789	1.105
Age	**<0** **.** **001**	1.119
iPSA	0.263	1.028

mpMRI PBx, mpMRI (multiparametric magnetic resonance imaging) targeted prostate biopsy; iPSA, initial PSA at diagnosis; DRE, digital rectal examination.

Bold values are statistically significant.

## Discussion

Even though there is a trend towards mpMRI PBx prior to surgery, as also observed in the current study, standard PBx is still the current standard of care in Germany (10, 21). The German Institute for Quality and Efficiency in Health Care (IQWiG) stated no evidence for the standardized use of an mpMRI PBx contrasting current guidelines (9, 10, 22). It's disheartening because, besides the established oncological advantage (21, 23), we had earlier demonstrated that a successful ns-RALP is predictably associated with an mpMRI-PBx (*p* < 0.001) (13). In the examined population SNR of neurovascular bundles occurred in 26% when PCa was diagnosed via mpMRI-PBx and in 56% for standard PBx (*p* < 0.001). A trend towards postoperative upgrading of the tumor after standard PBx suggests that standard PBx results sometimes underestimate PCa aggressiveness.

In the context of higher rates of successful ns RALPS in combination with prior mpMRI PBx (13), we hypothesized that patients in this setting could consequently experience improved preservation of erectile function. EPIC-26 questionnaire with a 1-year follow up was used to show the possible benefit of mpMRI PBx in (attempted) ns RALPs on functional outcomes. Concerning erectile function status, EPIC-26 seems to have more descriptive validity for not sexually active men compared to other instruments (24), especially for the difficult and interindividual assessment of ED (25). It is crucial to consider the preoperative erectile function for an accurate assessment of sexual function (25). Salonia et al. postulated that validated questionnaires with defined cut-offs, including the preoperative erectile function status, should be routinely used to enhance post-RALP satisfaction (25). According to the van der Slot findings (26), implementing the NeuroSAFE technique resulted in a continence rate of 92% at the 1-year mark and 94% at the 2-year mark among patients. Additionally, 44% of the men achieved a favorable or moderate score for erectile function at both, 1 and 2 years, following the surgery. In our multivariate analyses mpMRI PBX was found to be a predictor for a better erectile function 1 year following surgery. We found an almost 25% higher risk of suffering ED when PCa was diagnosed via standard PBx compared to patients with mpMRI PBx (*p* = 0.025).

We further observed less rates of SNR in the mpMRI PBx group compared to the standard PBx group (26.7% vs. 50.8%, *p* < 0.001). A discussion regarding the potential presence of a selection bias is necessary, taking into account the possibility of a more precise characterization of the carcinoma with mpMRI PBx compared to a standard PBx. Apart from iPSA the preoperative oncological patient characteristics for ns attempts did not differ from each other between the two groups. There were no differences in the preoperative histological- and clinical findings between the standard PBx and the mpMRI PBx population. However, the percentage of (attempted) nerve sparing out of all mpMRI PBx was 72% compared to 56% for standard PBx.

Successful ns-RALPs without SNR could be performed in 73% of the mpMRI PBx group and in 49% of the standard PBx group.

Interestingly, even though preoperative patient characteristcs did not differ between the two groups and fewer intraoperative SNR were needed in the mpMRI PBx group, a higher rate of final positive surgical margins (posSM) can be observed in the standard PBx group.

In our multivariate analyses, the risk of a PSM was more than twice as high when PCa was diagnosed via standard PBx compared to mpMRI PBx (*p* = 0.03). Also upgrading of the tumor was observed significantly more often in the standard PBx group. This fact, in addition to the lower SNR rate for mpMRI PBx in patients with (attempted) ns-RALPs suggests, that mpMRI PBx provides more, and correct information of the carcinoma and it seems that surgeons may be able to characterize the prostate and the carcinoma within the gland in a more precise way.

In our institution ns is standardly performed intrafascially. In a recently published review intrafascial ns showed advantages for urinary incontinence and EF compared to interfascial ns (2).

There are multiple different techniques to perform intraoperative frozen sections. Schlomm et al. firstly described the NeuroSAFE technique in 2012 (4). Beyer et al. transferred this frozen section technique into the RALP era (27). In our (attempted) ns RALPs we used NeuroSAFE to provide best oncological safety, since the “NeuroSAFE PROOF feasibility trial” states that no PSM seems to be missed in the NeuroSAFE intraoperative frozen section (5). Whenever an PSM was found intraoperatively we performed a full SNR of the whole neurovascular tissue on the adjacent side including the rectolateral half of the Denonvilliers fascia. Up till now SNR techniques are heterogenous. However, several studies confirm the usage of NeuroSAFE (4, 27–29).

In conclusion, the NeuroSAFE ns RALP procedure, even with the potential requirement for a full SNR does not appear to compromise the level of oncological safety.

To summarize our findings, we saw more ns attempts when mpMRI PBx diagnosed PCa with less SNR, a better functional outcome and less upgrading of the carcinoma postoperatively but with comparable preoperative conditions to standard PBx diagnosing PCa. We attribute the better erectile function to a lower rate of secondary resections of the neurovascular bundles and a better understanding of tumor spread in the gland. Therefore, a better functional result can be achieved by the surgeon. Although there was no selection bias towards less ns attempts in the mpMRI PBx group these findings suggest, that the oncological information combined with the imaging and knowledge of the intraprostatic distribution of carcinoma-lesions leads to a better understanding of the gland itself. This issue, however, remains speculative for our results but confirm existing studies (13, 23).

Finally, we observed a trend towards the usage of mpMRI-PBx prior to RALP (*p* = 0.001). While mpMRI PBx isn't currently a standard care procedure and comes with higher costs compared to standard PBx, studies have demonstrated its cost-effectiveness for the healthcare system. This is primarily due to the prevention of delayed diagnosis, understaging, biopsy-related complications, and unneeded repeat biopsies (30, 31).

Simultaneously to the increased usage of mpMRI-PBx we also saw a trend towards a higher rate of ns RALPs (*p* < 0.018), which could be explained by the increased usage of mpMRI-PBx. Another reason could be that the surgeons' experience increased over the years, and thus the proportion of nerve sparing RALPS did as well. But, at our clinic, during this period, there were 4 experienced surgeons (more than 100 surgeries) who performed the da Vinci surgeries. Only 1 surgeon was on his learning curve. Therefore, this effect is unlikely in our analysis.

This study completes our previously findings by adding follow-up data on erectile function (13). The biggest advantage of this study is the combination of functional and oncological data. The main limitations include the retrospective analysis of the prospectively collected data. However, prospective patient randomization would be largely unfeasible due to specific histological characteristics and patients' preferences. As such, we do not see the lack of randomization as a detrimental element of this study. However, we lack information about individual decision-making processes, such as whether ns was attempted or not. The next phase should involve assessing the impact of MRI on the surgical decision. Another limitation of our study was that the standard biopsy was performed by the outpatient urologist. Therefore, standardized execution is not guaranteed. However, this fact reflects the current care landscape in many parts of Germany.

## Conclusion

The combination of mpMRI PBx for PCa diagnosis followed by ns-RALP resulted in significantly fewer cases of SNR, better oncological outcomes and reduced incidence of ED 1 year after surgery. This included fewer PSM and a lower rate of postoperative tumor upgrading. Especially younger patients may potentially benefit from undergoing mpMRI PBx prior RALP. This approach not only contributes to improved oncological outcomes but also to the preservation of nerves to maintain erectile function.

## Data Availability

The raw data supporting the conclusions of this article will be made available by the authors, without undue reservation.
